# Improved soil carbonate determination by FT-IR and X-ray analysis

**DOI:** 10.1007/s10311-012-0380-4

**Published:** 2012-09-01

**Authors:** Viktor J. Bruckman, Karin Wriessnig

**Affiliations:** 1 Austrian Academy of Sciences (ÖAW), Commission for Interdisciplinary Ecological Studies (KIOES), Dr. Ignaz Seipel-Platz 2, 1010 Vienna, Austria; 2 Institute of Applied Geology, University of Natural Resources and Life Sciences, Peter Jordan-Strasse 70, 1190 Vienna, Austria

**Keywords:** Forest soil, Carbonate, Fourier transform mid-infrared spectroscopy, X-ray diffraction, Partial least squares regression, Scheibler method

## Abstract

In forest soils on calcareous parent material, carbonate is a key component that influences both chemical and physical soil properties and thus fertility and productivity. At low organic carbon contents, it is difficult to distinguish between organic and inorganic carbon, e.g. carbonates, in soils. The common gas-volumetric method to determine carbonate has a number of disadvantages. We hypothesize that a combination of two spectroscopic methods, which account for different forms of carbonate, can be used to model soil carbonate in our region. Fourier transform mid-infrared spectroscopy was combined with X-ray diffraction to develop a model based on partial least squares regression. Results of the gas-volumetric Scheibler method were corrected for the calcite/dolomite ratio. The best model performance was achieved when we combined the two analytical methods using four principal components. The root mean squared error of prediction decreased from 13.07 to 11.57, while full cross-validation explained 94.5 % of the variance of the carbonate content. This is the first time that a combination of the proposed methods has been used to predict carbonate in forest soils, offering a simple method to precisely estimate soil carbonate contents while increasing accuracy in comparison with spectroscopic approaches proposed earlier. This approach has the potential to complement or substitute gas-volumetric methods, specifically in study areas with low soil heterogeneity and similar parent material or in long-term monitoring by consecutive sampling.

## Introduction

Carbonate is a key component of soils on calcareous parent material, influencing both chemical and physical soil properties and hence fertility and productivity. It directly affects the soil pH and buffer capacity and is in strong reciprocation with biochemical cycles. Carbonate promotes the formation of stable soil aggregates and influences physical soil properties such as hydraulic characteristics and erodibility. It typically occurs as calcite (CaCO_3_) and/or dolomite (CaMg[CO_3_]_2_), depending on the parent material and soil formation processes. A number of different methods of carbonate determination in soils were proposed over the last decades, principally based on approaches in which released CO_2_ gas is directly measured either volumetrically or gravimetrically as a consequence of mass loss after treatment with acids. Other approaches are spectroscopic by means of Fourier transform infrared (FT-IR) or Raman spectroscopy or X-ray diffractometry (XRD) (Gunasekaran et al. 2006). A good overview of common methods is presented in Kamogawa et al. (2001). The gas-volumetric Scheibler method (ON L 1084-99, 1999) is still widely used, despite its weaknesses, for example, analytical precision, time consumption, and varying reaction times based on the present type of carbonates. Low solubility of dolomite in 10 % HCl leads to longer reaction times as compared to calcite (Schlichting et al. 1995), and consequently, total carbonate might be underestimated or organic carbon might be overestimated (after subtraction of inorganic carbon from total carbon). FT-IR spectroscopy, on the other hand, has proven to have the potential for accurate quantitative estimations (Janik and Skjemstad 1995; Tatzber et al. 2007, 2011; Ji et al. 2009). The current study aims at developing a model for carbonate quantification using a combination of FT-IR spectroscopy and XRD. Reference material is forest soil from a series of recently established long-term observation plots for studying woody biomass production. A similar approach was recently used in aquatic ecosystems for the identification of Heinrich events in marine deposits (Ji et al. 2009). We hypothesize that the determination of the form of carbonate actually occurring on our plots (calcite, dolomite) would increase the accuracy of predicting carbonate contents in our model.

## Experimental

### Soil sampling and preparation

The soil samples were collected in Eastern Austria, in a *Quercus* dominated high forest management system. The parent material consists of gravel, sand and silt built up during the Pannonium as a result of the early development of the Danube River. Parts of the deposited material originate from the northern limestone Alps, therefore containing considerable amounts of carbonate. Younger Aeolian deposits of loess (Pleistocene) from the same source region led to periglacial formation of Chernozems. A detailed site description and soil characterization as well as belowground organic carbon dynamics can be found in Bruckman et al. (2011). Only soils that obviously contained carbonates (determined by a pH [CaCl_2_] value > 6 (Cools and de Vos 2010)), which is the case for approximately 10 % of all samples, were selected for this study. As a consequence of the geological background and diverse parent material, both calcite and dolomite are present in the soil matrix. Three soil reference groups were identified according to WRB (IUSS Working Group WRB 2006) across our study region, where five plots are classified as Cambisols, three as Luvisols and one as Chernozem. Mineral soil cores were divided into five geometric horizons, 0–5, 5–10, 10–20, 20–40 and 40–50 cm soil depth, and were analysed separately. Soil samples were sieved to 2 mm, and a subsample was ground to powder for Fourier transform mid-infrared (FT-MIR) and XRD analysis.

### Gas-volumetric Scheibler method

The gas-volumetric determination of carbonate (Scheibler method) was conducted following ÖN L 1084-99 (1999), also described in Tatzber et al. (2007). Carbonate contents calculated from the Scheibler method were corrected for the dolomite content of the respective samples using a stoichiometric coefficient based on the calcite/dolomite XRD peak area ratios. Ambient air pressure and temperature were recorded for each sample in order to calculate the pT coefficient, which corrects the volume of CO_2_ for current temperature and air pressure during the reaction. The reaction time was extended in order to allow complete dissolution of dolomite in the samples.

### Fourier transform mid-infrared spectroscopy

**Fig. 1:**
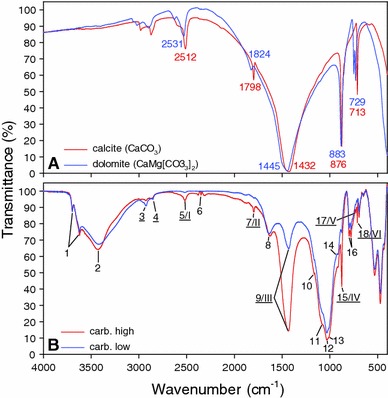
Pure carbonate and bulk soil infrared spectra. **A** Mid-infrared (MIR) range spectrum of pure calcite (CaCO_3_) and dolomite (CaMg[CO_3_]_2_). Calcite and dolomite peaks used in this study are indicated by their respective wavenumber. **B** MIR spectra of bulk soil samples with the lowest (carb. low) and the highest (carb. high) carbonate concentrations of all samples. Carb. high: 19.6 % carbonate by weight, 40–50 cm soil depth, calcite/dolomite ratio = 1.52, Chernozem. Carb. low: 0.5 % carbonate by weight, 0–5 cm soil depth, calcite/dolomite ratio = 0.92, Chernozem. Peaks are indicated for most important infrared-active functional groups occurring in mineral soils (Tatzber et al. 2010). Indicative peaks for carbonate are *underlined,* and *Roman numbers* represent peaks used in our model. 1 = Si–O–H vibrations of layer silicates; 2 = O–H (H bonded), N–H; 3 = asymmetrical CH_2_ stretching, carbonate; 4 = symmetrical CH_2_ stretching, carbonate; 5 = carbonate; 6 = ambient CO_2_; 7 = carbonate (calcite dominated); 8 = C–O stretch (carboxylates, aromatic vibrations), O–H bending of water; 9 = carbonate, carboxylates; 10 = C–O stretch of OH deformation of COOH; 11 = Si–O–Si, sulphate; 12 = C–O stretch, carbohydrates; 13 = SiO_3_^2−^, CH vibrations; 14 = C–H deformation of aromatics, cyclopentane; 15 = carbonate; 16 = quartz; 17 = dolomite; 18 = calcite

KBr pellets were produced to measure samples in transmission mode in the mid-infrared area, that is, 400–4,000 cm^−1^, using a Bruker Optics Tensor 27 spectrometer. Approximately 1 mg bulk soil sample, which was dried at 70 °C for at least 12 h prior to measurement, was mixed with 200 mg FT-IR grade KBr (both weighed exactly) and ground in an agate grinding mill. The mixed bulk soil and KBr powder was immediately pressed to obtain a transparent pellet and measured. According to our observation, it is crucial to store KBr and all manipulation tools such as press chambers and cylinders at 70 °C as this promotes sintering during pressing and results in completely transparent pellets of high quality. Sixteen scans were performed for each sample and averaged. The spectra were corrected against a pure KBr pellet and ambient air. A background spectrum was measured at least every 30 min. All spectra were baseline corrected before conducting further analysis. According to Beer’s law, the magnitude of a signal is proportional to the concentration of the respective component causing IR absorption at its specific wavelength, allowing quantitative conclusions. For this reason, absorption spectra were used for analysis, rather than transmission spectra that are shown in Fig. 1. Six indicative peak regions were selected for integration with base points at 2,686 and 2,460 cm^−1^ (Peak I), 1,850 and 1,784 cm^−1^ (Peak II), 1,567 and 1,295 cm^−1^ (Peak III), 889 and 867 cm^−1^ (Peak IV), 734 and 719 cm^−1^ (Peak V), and 719–708 cm^−1^ (Peak VI). Location of peaks was derived from Ji et al. (2009) and Tatzber et al. (2010). While peaks I–IV represent a combination of calcite and dolomite, which is not clearly separable, V is indicative of dolomite and VI of calcite. The exact location of the peaks and base points was set according to our own results; hence, they are not necessarily convergent to the cited papers.

### Powder X-ray diffraction

Powder XRD was performed with ground bulk soil samples using a PANalytical X’pert Pro Diffractometer with CuKα radiation at a tension of 45 kV, and a current of 40 mA. Scanning was performed from 5° to 70°2θ at an interval of 0.017°, with a measuring time of 25 s per step. X-Ray diffraction allows a distinct differentiation between calcite and dolomite and calculation of respective ratios, which could be used to raise accuracy of carbonate determination. Approximately two grams of ground sample material is needed, which can be recycled and used for further analysis since the measurement is non-destructive. Sample preparation is simple and measurement is relatively fast if newer instrumentation is used (~15 min per sample) and no additional consumables are needed. However, the main limitations are its detection limit which is around 1 % by weight and relatively high investment costs for the analytical equipment (Ji et al. 2009). Two indicative peaks were selected for integration, representing calcite at 29.5°2θ (Peak VII) and dolomite at 31°2θ (Ji et al. 2009). Base points were set individually for each sample, representing local minima before and after the peak location.

### Partial least squares regression

Partial least squares regression (PLSR) was used to develop a model for carbonate estimation at our study site. PLSR is among the most commonly used procedures for spectral chemometrics, which is also confirmed in the comprehensive review by Rossel et al. (2006). The first step is a reduction in dimensions. Principal components are computed from the predictor values, which are used to predict scores of the dependent values’ components. Subsequently, the scores are used to compute the actual dependent values. Advantages of this method are its relative robustness with respect to missing data and noise and its ability to handle multicollinearity among the independent input values. The statistical software R (R Development Core Team 2011) in combination with the PLS package (Mevik and Wehrens 2007) was used to develop our model.

## Results and discussion

### Results obtained by the Scheibler method

Using the Scheibler method, we found a mean carbonate content of 5.4 % by weight, ranging from 0.5 to 19.6 %, which represents contents of 4.6–196.4 mg g^−1^. The mean calcite/dolomite ratio, based on the integrated XRD bands VII and VIII, was 1.18, when excluding four samples with low dolomite contents which present an elevated mean ratio of 41.67. The four samples were from the plot where the soil was identified as Cambisol. In situ observations during soil sampling showed clear signs of secondary carbonates, that is, carbonates that are formed by biological processes at these rather dry sites and consist of calcite. Earthworm-secreted carbonate could be a significant component of biogeochemical C and Ca cycling (Lambkin et al. 2011). Based on this observation, grinding the soil samples for analysis is strongly suggested after sieving (2 mm).

### Combination of Fourier transform mid-infrared and X-ray diffraction results

Pure calcite and dolomite spectra are shown in Fig. 1A. Bands appeared at 2,984, 2,873, 2,512, 1,798, 1,432 cm^−1^ (ν_3_), 876 cm^−1^ (ν_2_), and 713 cm^−1^ (ν_4_). Tatzber et al. (2007) produced very similar results on pure calcite, using a different instrument (Perkin Elmer Paragon 500 Spectrometer), and a detailed description of the underlying functional groups of the respective bands can be found in their study. It seems that dolomite peaks appear at shorter wavelengths, with a mean difference of 21.4 ± 11.1 cm^−1^ in comparison with calcite. Respective dolomite bands appeared at 3,024, 2,902, 2,531, 1,824, and 1,445 cm^−1^ (ν_3_), 883 cm^−1^ (ν_2_), and 729 cm^−1^ (ν_4_). This comparison reveals potential difficulties in setting appropriate base points when integrating bands of bulk soil samples since carbonate typically occurs in different relative amounts of calcite and dolomite (sometimes also other carbonates, e.g. siderite) in soils. As a consequence, bands of bulk soil samples (Fig. 1B) are less distinctive and usually broader as compared to pure carbonate spectra and in some cases overlapped by other non-calcareous constituents. This, for instance, in the 3,024–2,873 cm^−1^ band area, which is overlapped by aliphatic groups, causes CH_2_ stretching signals. Hence, this area was excluded for carbonate determination based on FT-MIR spectroscopy. We selected an integration mode where the integrated peak area (corrected for the amount of sample) is delimited by the peak itself within the base points and a straight line between them. Integration up to the *x*-axis showed weaker model performances, which was most likely caused by slightly different amounts of sample in each pellet.

While most bands appear in the same magnitude at a given amount of sample, some bands tend to show weaker signals in case of dolomite, which is especially true for the 1,824 cm^−1^/II band. If only this band is considered for predictive calculations and treated as a common carbonate band, one might underestimate carbonate if the soil contains considerable amounts of dolomite.

**Fig. 2:**
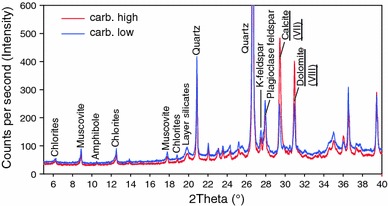
X-ray diffractogram of the same carb. high and carb. low bulk soil samples as shown in Fig. 1B. The most important peaks to characterize the soil are indicated. Note the distinct separation of calcite and dolomite

X-ray diffractograms (Fig. 2) reveal a distinct pattern of mineral soil samples, with clear indicators of considerable amounts of clay minerals (e.g. chlorites) in general and to some extent, more specific details of other types of layer silicates. Being one of the fractions most resistant against chemical weathering in soils, quartz represents the most dominant peaks at 20.9°2θ and 26.6°2θ. Calcite (at 29.5°2θ/VII) and dolomite (at 31°2θ/VIII) are well separated and clearly no overlap occurs, making it comparatively simple to set base points for peak area integration and to determine the calcite/dolomite ratio to improve the Scheibler results. Using this approach, our results were corrected by up to 3.8 %, while the mean correction factor was 0.1 %, depending on the calcite/dolomite ratio and the pT factor during Scheibler measurement.

**Fig. 3:**
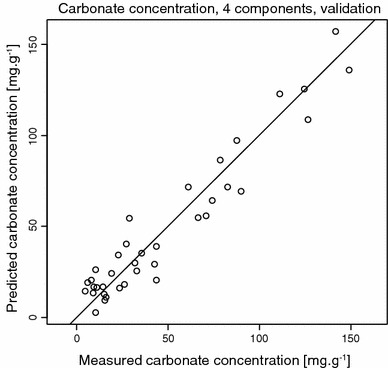
Cross-validated predictions for carbonate contents derived by Scheibler method, corrected for calcite/dolomite ratios. The lowest root mean squared error of prediction (RMSEP (r_CV_^2^) = 11.57) (adj. CV) was derived with a model including four principal components and XRD calcite and dolomite peaks in combination with improved Scheibler carbonate estimates. Cross-validation training explained 100 % of the variance of predictors and 94.5 % of the variance of the dependent (carbonate concentration)

Figure 3 shows the results of the carbonate prediction model including mid-infrared bands and the calcite and dolomite peaks from XRD as predictors and corrected Scheibler carbonate concentrations as the dependent variable; 94.65 % variance of the predictors was explained, employing a model that embraces four principal components. Including more components leads to a higher root mean squared error of prediction and hence a poorer model performance as a consequence of increased model complexity. Our model represents the data set satisfactory, and both inclusions of bands VII and VIII from XRD and the improved Scheibler estimates (stoichiometric coefficient) enhanced overall performance. As a result, the root mean squared error of prediction decreased from 13.07 to 11.57. The improvement was pronounced in samples with higher carbonate contents >100 mg g^−1^. However, we had to exclude one sample with the highest carbonate content (196.4 mg g^−1^) from our model calculation as it turned out to be an outlier.

### Interpretation of main model components

**Fig. 4:**
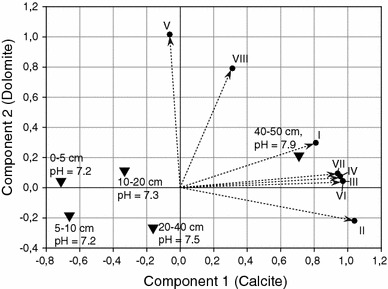
Results of a principal component analysis (PCA) with oblique rotation at KMO = 0.877 and Bartlett’s test of sphericity χ^2^ (28) = 755.84; *p* < 0.001. Component loadings of mid-infrared and XRD bands are shown (*black dots*, see Experimental section for band assignments and wavelength information) together with genetic soil horizons (*triangles*) and their respective pH value (H_2_O)

Calcite and dolomite represent the first two main components, and a loading plot is shown in Fig. 4. While bands I–IV, VI, and VII are indicative of calcite or calcite and dolomite and load predominantly on Component 1, bands V and VIII represent dolomite and predominantly load on Component 2. Depicted positions of soil horizons with their respective pH value in aqueous solution clearly show the trend of increasing carbonate contents and pH values with increasing soil depth. However, it seems that calcite is the main determinant of this observation, which could be explained by its relatively better solubility in weak acids, such as precipitation and humic acids in soil solution as compared to dolomite; hence, it is preferably translocated into deeper soil horizons in forest soils. This illustrates the importance of considering soil inorganic carbon as a combination of different forms of carbonate, influencing soil chemical processes, and utilizing quantitative spectroscopic approaches, and examples from previous research in marine sediments (Ji et al. 2009) underline this proclamation.

## Conclusion

For the samples in our study from three different soil reference groups, the model shows a good performance in predicting carbonate contents across the study region. The proposed method can be used as an alternative approach for estimating carbonate content in soils. Here, we show that PLSR is a suitable method, even if the sample size is small, and that the model performance is increased by including information on the actual occurring forms of carbonate, for example, bands V and VII as a proxy for dolomite. As compared to other published spectroscopic approaches, based typically on a single method, a combination of methods has the potential to improve accuracy. However, we suggest a larger study, based on more samples from different soils and vegetation types to validate our results. Samples with high carbonate contents (>15 %) should be included to test the stability of the proposed method at high concentration levels.
